# TCM practitioners' perspectives on AI-assisted *ad hoc* interpreting in China: a qualitative study

**DOI:** 10.3389/fpubh.2026.1939217

**Published:** 2026-10-01

**Authors:** Bowen Geng, Shan Zhu

**Affiliations:** 1 School of International Studies, Shandong Youth University of Political Science, Jinan, China; 2 School of International Journalism and Communication, Beijing Foreign Studies University, Beijing, China; 3 School of Interpreting and Translation, Beijing International Studies University, Beijing, China

**Keywords:** *ad hoc* interpreters, AI, cross-linguistic healthcare, health equity, interpreting, TCM practitioners

## Abstract

**Objective:**

Healthcare providers' perspectives on interpreting services offer critical insights for interpreters training and policy development. In China, AI-assisted *ad hoc* interpreting arrangements are the status quo; however, little is known about practitioners' perspectives on this mode in cross-linguistic TCM (Traditional Chinese Medicine) clinical practice. This study is part of an attempt to bridge that gap.

**Methods:**

A cross-sectional qualitative study design was employed. Semi-structured interviews were conducted with 21 TCM practitioners, and the data were analyzed using thematic analysis.

**Results:**

Six themes were identified from the data: information loss and diagnostic uncertainty; when equivalence fails, inequivalence emerges; redistribution of power and trust relations; communication barriers and professional identity erosion; necessary but suboptimal; and beyond interpreting toward understanding.

**Conclusions:**

TCM practitioners reported considerable drawbacks in using “*ad hoc*+AI” mode, with implications for clinical compromise and erosion of professional identity. There is a need for professional interpreting services to ensure that all individuals seeking TCM care can receive equitable care.

## 1 Background

Healthcare outcomes are closely tied to provider-patient communication (1). Communication breakdowns due to language barriers frequently diminish patients' care experience and treatment adherence, and may give rise to adverse clinical outcomes (2–4). As global migration continues to grow, cross-linguistic healthcare encounters are becoming more prevalent, resulting in a parallel rise in the demand for medical interpreting services (5). Professional interpreters are broadly considered as the preferred approach to overcome language barriers, as they can substantially improve communication quality, reduce clinical errors, and enhance patient satisfaction (6, 7). Nevertheless, owing to constraints in funding, scheduling, and institutional capacity, *ad hoc* interpreters without systematic training—including patient's family members and acquaintances, bilingual healthcare providers, or administrative staff—have become the most common alternative means of communication (8–10). In recent years, the rapid evolution of AI technologies has opened up new opportunities for healthcare interpreting services, with neural machine translation and AI translation tools being increasingly applied in medical encounters (11–13).

With the deepening of globalization and the increasing frequency of international exchanges, the number of internationals with limited Chinese proficiency coming to China for work, study, tourism, or residence has been continuing to grow. According to China's National Immigration Administration, the volume of foreign entries and exits in China was as high as 82.035 million person-trips in 2025 (14), they may also require healthcare services during their stay, and some choose to seek TCM treatments. TCM possesses a distinctive theoretical system and diagnostic and therapeutic approach that differ substantially from those of Western medicine, which is more familiar to most international patients (15, 16), thereby posing additional challenges for healthcare interpreting. China has not yet developed a standardized professional healthcare interpreter certification, training, and dispatch system at the national or industry level (17). This lack of institutional infrastructure has led local hospitals and medical institutions to rely extensively on *ad hoc* interpreters when dealing with language barriers (18). At the same time, AI tools are increasingly being employed by *ad hoc* interpreters in healthcare interpreting in China. In March 2026, preliminary telephone inquiries with 20 Class III Grade A public hospitals in four major metropolitan cities in China (Beijing, Shanghai, Guangzhou and Hangzhou) suggested that none routinely provided professional interpreting services for international patients. Instead, hospital staff indicated that AI-assisted *ad hoc* interpreting was the most commonly used interpreting mode. This mode is hereafter referred to as the “*ad hoc*+AI” mode.

As the primary recipients of healthcare interpreting services and the ultimate decision-makers in medical care, healthcare providers' perceptions and evaluations of interpreting provide important evidence for interpreter training and language service policy development (19). While there is a body of research exploring the healthcare providers' perspectives in countries with large immigrant populations (19–22), research in China remains limited, and no research has yet addressed the particular mode of “*ad hoc* + AI” in the context of TCM. In light of this, we conducted semi-structured interviews with 21 TCM practitioners to explore their perspectives toward “*ad hoc*+AI” mode to further examine the underlying factors and potential impacts, and investigate their expectations of ideal interpreting services. By doing so, we seek to fill this knowledge gap, provide evidence for improving healthcare experience of international patients, and contribute to equitable healthcare access.

## 2 Methods

This study formed part of a larger project approved by the Institutional Review Board (IRB) of Shandong Youth University of Political Science (IRB 2026012). We recruited 21 TCM practitioners through snowball sampling (15 men, 6 women), aged between 37 and 52 years (M = 45.05, SD = 5.01). They were all from Class III Grade A hospitals or international hospitals in China's first-tier cities. We sought to maximize sample variability through recruiting participants from diverse backgrounds, the demographic information for each participant is shown in Table 1.

**Table 1:** Participant information.

Participant	Gender	Age	Years in practice	Institution	Professional title	Clinical areas of expertise
A	37	Female	6	Class III Grade A hospital	Attending Physician	Women's health
B	45	Male	14	Class III Grade A hospital	Associate Chief Physician	Cardiovascular diseases Rehabilitation and pain management
C	51	Female	26	Class III Grade A hospital	Associate Chief Physician	Reproductive health
D	40	Male	9	International Hospital	Attending Physician	Complex and refractory conditions
E	48	Female	16	International Hospital	Associate Chief Physician	Respiratory diseases
F	42	Male	10	Class III Grade A hospital	Associate Chief Physician	Women's health
G	46	Female	13	International Hospital	Associate Chief Physician	Skin disorders
H	50	Female	24	Class III Grade A hospital	Chief Physician	Spleen-stomach disorders
I	39	Female	7	International Hospital	Associate Chief Physician	ENT disorders Allergic and respiratory conditions
J	44	Male	15	International Hospital	Associate Chief Physician	Geriatric conditions
K	48	Male	23	Class III Grade A hospital	Associate Chief Physician	Endocrine disorders Anxiety and stress-related conditions
L	52	Male	28	Class III Grade A hospital	Chief Physician	Gout Spleen-stomach disorders
M	46	Male	18	Class III Grade A hospital	Associate Chief Physician	Men's health
N	38	Male	6	International Hospital	Associate Chief Physician	Preventive Treatment of Disease
O	44	Male	12	Class III Grade A hospital	Associate Chief Physician	Respiratory diseases
P	50	Male	25	Class III Grade A hospital	Chief Physician	Integrated management of complex conditions
Q	41	Male	8	Class III Grade A hospital	Associate Chief Physician	Chronic disease management
R	47	Male	13	Class III Grade A hospital	Associate Chief Physician	Anxiety and stress-related conditions Chronic fatigue and functional disorders
S	39	Male	7	Class III Grade A hospital	Associate Chief Physician	Metabolic disorders Postoperative rehabilitation
T	43	Male	10	International Hospital	Associate Chief Physician	Health maintenance and preventive care
U	49	Male	16	International Hospital	Associate Chief Physician	Complex and refractory conditions

The first author conducted one-on-one interviews via the online conferencing platform Tencent Meeting, and all participants signed informed consent forms electronically prior to the interviews. The interviews were conducted in Chinese and the topic guide is outlined in Table 2. All participants reported treating international patients within the past 6 months using the “*ad hoc*+AI” mode, and *ad hoc* interpreters used general-purpose AI tools such as ChatGPT, DeepSeek, and Doubao to assist with interpreting. Before the formal interview, we conducted 2 pilot interviews and made minor adjustments to the interview protocol based on the results. We kept recruiting participants until data saturation was reached. All interviews were audio-recorded. Each interview lasted 45–95 min.

**Table 2:** Interview protocol.

Interview questions for TCM practitioners
1. What typically happens when an international patient comes into your clinic? (Please describe a typical consultation process.) How, if at all, this process differs from seeing a Chinese patient? 2. Can you describe an especially memorable experience in which communication with a foreign patient was particularly successful or particularly difficult? What happened at the time, and how did you feel about it? 3. In cross-linguistic clinical practice, what do you consider the most critical stage of the process (e.g., building trust, accurate syndrome differentiation, explaining pathogenesis, ensuring treatment adherence)? Why? 4. In communication, what is the greatest challenge? Is it the language barrier, difficulty in explaining TCM terms or something else? 5. Who would help you communicate with international patients? When professional interpreters are unavailable, who typically interprets for you? Do they use AI to assist with interpreting, if yes, which AI tool(s) do they typically use? 6. In what ways does AI-assisted *ad hoc* interpreting address the communication challenges discussed earlier? What new challenges emerge? 7. How have these challenges affected your clinical decision-making, treatment outcomes, or your own professional experience and professional identity? What is the biggest difference compared with consultations without AI assistance? 8. When interpreting errors or ambiguities occur, who usually notices them first? How does this affect trust and the power dynamics between the physician and the patient? 9. Setting aside current cost and technological constraints, what would your ideal form of cross-linguistic and cross-cultural interpreting service in TCM clinical practice look like? What core functions should it have? What would be the biggest difference compared with current practice? 10. What do you think hospitals, academia, or policymakers need to do in order to genuinely support TCM practitioners in conducting more effective cross-cultural clinical practice?

The first author transcribed the interview data, the corresponding author verified the transcripts. Subsequently, two authors independently analyzed the transcribed data using reflexive thematic analysis in the coding software NVivo 12, following the procedures proposed by Crowe et al. (23). First, the transcripts were read repeatedly to generate initial themes. Themes were clustered into related groups of ideas and further defined and named. Second, quotations were selected from the data to illustrate and support the core meaning of each theme. Third, through repeated writing and revision, each theme was further analyzed in depth and the relationships among the themes were examined. Fourth, the relationship between themes and the broader sociocultural context was examined. Lastly, two researchers reviewed and discussed the coding and analytical results until consensus was reached. To protect the data confidentiality and participants' privacy, all transcripts were anonymized and all participants were assigned alphabetical codes.

## 3 Results

This section presents TCM practitioners' perspectives on “*ad hoc*+AI” mode. Examples of verbatim extracts from the interviews are included to illustrate and substantiate research findings. Six themes can be identified from the data: information loss and diagnostic uncertainty; when equivalence fails, inequivalence emerges; redistribution of power and trust relations; communication barriers and professional identity erosion; necessary but suboptimal; and beyond interpreting toward understanding.

### 3.1 Information loss and diagnostic uncertainty

TCM practitioners generally reported that the “*ad hoc* + AI” mode led to the loss and deviation of information, thereby potentially affecting syndrome differentiation.

First, under the “*ad hoc* + AI” mode, patients' originally rich expressions were often compressed into simplified or even fragmented information, which made it difficult for practitioners to obtain sufficient information to support accurate syndrome differentiation. This is expressed in Excerpt 1:

*At times, the patient would say a great deal to me, yet the (ad hoc) interpreter produced only a short sentence, with a clear mismatch in length and amount of information. As a result, I simply did not have sufficient information to conduct syndrome differentiation. Many (ad hoc) interpreters believe their English is quite good, but they are unable to handle TCM healthcare interpreting. (Practitioner T)*

Second, practitioners were often unaware of what information had been omitted during the interpreting process, as shown in Excerpt 2:

*Sometimes the (ad hoc) interpreters would search with AI while interpreting, but more often they rendered everything into only a few words, leaving me unable to know what had been filtered out. (Practitioner E)*

Third, *ad hoc* interpreters were often unable to reproduce patients' tone, emotional reactions, and ways of expression, making it difficult for practitioners to evaluate patients' emotional states or the severity of their conditions through tone and emotional expression as they would when directly treating Chinese-speaking patients. This can be seen in Excerpt 3:

*I need to assess patients' emotional state by observing their facial expressions and tone of voice—whether they speak in a high-spirited, excited manner or in a low, listless one is a key area of examination. However, this mode fails to convey these aspects. Many times, the patients and ad hoc interpreters would whisper to each other, and ad hoc interpreters would also fail to convey patients' emotions; or they would simply let AI interpret, and AI was always like a robot in terms of tone, leaving no way for me to make any assessment. (Practitioner J)*

Finally, participants reported that *ad hoc* interpreters had not received systematic training and were unable to master TCM healthcare interpreting, while AI translation tools also could not ensure interpreting accuracy; therefore, this mode could lead to information distortion and consequently result in diagnostic misjudgments during medical consultations. This is reflected in Excerpt 4:

*Although they can speak English, they still have problems with expressions in medical aspects and even in daily life. Although they had AI tools, errors still occurred in the interpretation; on one occasion, an AI tool even mistranslated urination and defecation. Consequently, this may also result in practitioners making an incorrect diagnosis based on incorrect information. (Practitioner K)*

### 3.2 When equivalence fails, inequivalence emerges

Practitioners further reported that communication difficulties stemmed not only from information loss, but more fundamentally from the structural mismatch between the underlying medical frameworks of TCM and Western medicine.

First, some *ad hoc* interpreters and AI translation tools would aligned TCM terms with “equivalent terms” from Western medicine, even though the two were not fully equivalent. This approach facilitated initial understanding to a certain extent, but in fact altered the theoretical foundation of the original concepts, causing conceptual interpretation to shift from the TCM framework to the biomedical framework. As a result, some patients were more likely to prefer highly targeted, fast-acting interventions while questioning gradual, holistic approach of TCM. They frequently displayed uncertainty or hesitation, thereby potentially compromising treatment adherence. This is expressed in Excerpt 5:

*Some ad hoc interpreters and AI tools use Western medicine terms to interpret TCM terms, and they may do so without being aware of the differences between the two and thus take it for granted, which leads the patients to also understand it from the perspective of Western medicine. For example, some ad hoc interpreters and AI tools may render “*血虚”* (Xue Xu) as the Western medical term “anemia”. However, in TCM, “*血虚”* or “blood deficiency” refers to insufficient nourishing function of the body's blood; whereas “anemia” in Western medicine is defined as hemoglobin or red blood cells in the blood dropping below a specified threshold. Thus, the patients may think: since the practitioner says I have anemia, I can just go to my family doctor and get some iron supplements, why would I still bother to take such bitter Chinese herbal medicine? (Practitioner O)*

Second, even when *ad hoc* interpreters and AI rendered certain TCM terms literally, the meanings that practitioners and patients attached to the same term did not fully align, resulting in a form of communication inequivalence characterized by surface alignment but deep misalignment. Practitioners' discourse in the TCM context could be re-encoded within patients' existing cognitive frameworks, hindering mutual comprehension between the two parties. This is illustrated in Excerpt 6:

*Sometimes, even with the assistance of AI, (ad hoc) interpreters' word-for-word translation fails to resolve the issue. It is unable to convey the intended meaning and may even lead to significant misunderstandings, as many TCM terms lack equivalent concepts in Western medicine. Take “*胃火炽盛”* (Wei Huo Chi Sheng) for example, AI interprets it literally into “you have excessive stomach fire”, then the patient would be scared out of his wits just by hearing that and suspect that this practitioner is talking nonsense. (Practitioner H)*

### 3.3 Redistribution of power and trust relations

With the involvement of *ad hoc* interpreters and AI, trust and power were redistributed from the “practitioner-patient” dyad into a multi-party negotiation among practitioner, *ad hoc* interpreter, AI, and patient. Practitioners' control over information and the level of trust between providers and patients were challenged.

In the “*ad hoc*+AI” context, *ad hoc* interpreters controlled the flow of information and could filter, simplify, and replace the information exchanged between the practitioner and patient, or even directly take over the conversation and speak on behalf of the patient. Both the practitioner and the patient, who originally occupied the core positions in the interaction, became marginalized, as illustrated in Excerpt 7 and Excerpt 8 respectively:

*Sometimes the interpreter and the patient chatter on and on, but the interpreter doesn't interpret for me. Other times, I say quite a bit to the interpreter, and he or she turns around and says just a few short sentences to the patient. This means that what the patient and I can know depends entirely on the interpreter, and my own sense of control over the communication is weakened. (Practitioner D)* *A very common situation is: I ask the patient a question, but the ad hoc interpreter doesn't interpret it — instead, he or she answers directly on the patient's behalf, and the patient gets sidelined. This happens especially when the interpreter is the patient's family member. (Practitioner I)*

Some practitioners reported that frequent interpretation errors, distortions, or information omissions produced by “*ad hoc*+AI” mode affected the establishment of trust between practitioners and patients to a certain extent. When patients repeatedly found discrepancies in the communication, they would begin to doubt whether practitioners truly understand them, and subsequently questioned practitioners' professional competence; meanwhile, practitioners would worry that patients had not truly understood their diagnostic and therapeutic intentions. This unstable communicative state further made it difficult for both parties to establish a stable therapeutic relationship, as expressed in Excerpt 9:

*When interpretation errors occur repeatedly, patients may begin to doubt me, believing that I do not truly understand them or that my professional competence is insufficient to support an accurate understanding of their condition. In such circumstances, how can patients possibly trust me? And how can treatment adherence be established? (Practitioner F)*

Some practitioners further stated that patients placed excessive trust AI-generated interpretations. When the AI's interpretation conflicted with practitioners' views, some patients were more likely to believe the AI rather than practitioners' professional judgment, as presented in Excerpt 10:

*Some patients place blind trust in AI. If I do not agree with the AI, they think I am wrong. I remember that a translation app translated the TCM term “*上火”* as “inflammation”, which is clearly not the same thing. But this idea had already been preconceived by the patient, and he kept asking me whether he should take antibiotics. I had to spend a great deal of effort explaining that the two are not the same before he could finally trust what I said. (Practitioner L)*

In addition, some practitioners reported “*ad hoc*+AI” mode compromised confidential communication between them and patients, raising concerns about confidentiality and ethics. They stated that due to concerns that their private information might be disclosed, some patients deliberately withheld information when discussing sensitive content, which weakened the trust between two parties and also affected patients' ability to express themselves openly as well as practitioners' ability to conduct in-depth consultations. This is expressed in Excerpt 11 and Excerpt 12:

*Sometimes patients are accompanied by friends or colleagues, and when relatively private or sensitive issues are raised—such as emotions, family relationships, genital area, or sex-related topics—they become concerned that their privacy may be disclosed to others and are reluctant to speak. We also find it difficult to ask further questions, and as doctors, we therefore fail to obtain these key pieces of information. (Practitioner C)* *Some patients place great importance on their privacy and do not fully trust AI, worrying that their information may be collected by big data. As a result, they tend to avoid answering questions when it comes to sensitive or private issues. (Practitioner G)*

### 3.4 Communication barriers and professional identity erosion

In cross-linguistic clinical encounters involving “*ad hoc*+AI” mode, persistent communication difficulties altered practitioners' clinical practice and professional experience, and eroded their professional identity.

First, to facilitate communication, some practitioners deliberately reduced TCM theoretical explanations and even used expressions more consistent with Western medical terminology. Although this compromise helped improve communication efficiency, the educational functions in TCM clinical practice were significantly weakened. Excerpt 13 highlights this issue:

*Even if much more is said, it still cannot be properly interpreted through “ad hoc*+*AI” mode, and there is a risk of increasing distortion or errors. As a result, diagnosis becomes more difficult, and patients may grow more suspicious of me, and even of TCM. So I try to say as little and “simple” as possible, and I stop saying certain things that I would normally say to Chinese patients. However, in doing so, patients' understanding of TCM, as well as their trust in and adherence to practitioners' recommendations, may be also compromised. (Practitioner R)*

Second, because the content of the consultation, when relayed through “*ad hoc*+AI” mode, was prone to omissions, simplification, or distortion, practitioners conducted less complex follow-up questioning and relied more on tongue and pulse examination to make clinical judgments. As a result, practitioners tended to adopt relatively “safe” and broadly applicable treatment plans, rather than highly individualized, syndrome-based interventions, which is described in Excerpt 14:

*In TCM, diagnosis is based on the four methods of observation, listening/smelling, inquiry, and palpation. Under such circumstances, the inquiry component is often difficult to complete comprehensively, and even the full set of the “Ten Questions” (Shi Wen Ge*, 十问歌*) and the necessary follow-up questions may not be fully covered. Some subtle and fine-grained questions can no longer be asked, and ultimately practitioners have to rely mainly on pulse-taking and tongue diagnosis, prescribing relatively mild and conservative formulas to avoid risk, although this may reduce the therapeutic effect. I know this is not ideal, but there is no real alternative. (Practitioner A)*

Last, some practitioners further noted that prolonged “simplification” and “compromise” could gradually affect their professional experience. Practitioners were not able to fully conduct syndrome differentiation due to limitations of “*ad hoc*+AI” mode, which led some respondents to feel that their professional value could not be fully demonstrated, and it in turn weakened professional identity for practitioners. Excerpt 15 provides an example of this:

*In such situations, in-depth communication is often difficult to achieve, and prescribing a formula that appropriately matches the patient's condition may also be challenging, thereby potentially compromising treatment effectiveness. Patients may think that TCM is not as effective as expected or as commonly believed. I also feel that professional identity is diminished. Prolonged exposure to this state can lead to job burnout. Seeing international patients makes me feel tired—not physically, but rather because I know the situation cannot be effectively managed, yet there is nothing I can do about it. (Practitioner S)*

### 3.5 Necessary but suboptimal

Although most practitioners noted a range of problems with the “*ad hoc*+AI” mode, most of them still regarded it a necessary compromise in the current healthcare context.

Due to issues such as cost and accessibility, professional interpreting services were much less available than “*ad hoc*+AI” mode, particularly during emergency care, nighttime consultations, or outpatient peak hours, practitioners were often unable to find suitable professional interpreters in a timely manner, whereas the “*ad hoc*+AI” mode was highly accessible and thus became a default alternative. This is expressed in Excerpt 16:

*It's not as if I refuse to employ professional interpreters; the trouble is that there just aren't any available. The hospitals don't hire any, and patients are either unwilling to bear the expense or even unable to access one. So what other choice do I have? For now, I just get by with what I have—it's better than nothing at all. (Practitioner D)*

Some practitioners further reported this originally temporary practice was gradually evolving into the default mode in everyday clinical care, as illustrated in Excerpt 17:

*Over time, it gradually became routine practice. Although the hospital administration realized it was far from ideal, they still accepted it by default because there were no other options. (Practitioner U)*

Most practitioners still viewed this mode as merely a suboptimal solution under current circumstances rather than an ideal approach to cross-cultural clinical practice, as reflected in Excerpt 18:

*This mode is certainly far from ideal, and I am definitely not satisfied with it, but for now this is the only option. Hopefully, professional interpreters will be available in the future. (Practitioner M)*

### 3.6 Beyond interpreting toward understanding

Many practitioners reinforced that effective communication involved more than merely “accurate and complete interpreting”; it required allowing patients to fully understand the logic of syndrome differentiation and treatment of TCM.

First, practitioners stated that an ideal interpreter should be primarily capable of delivering information accurately and completely, which calls for solid interpreting competence and professional ethics. This is expressed in Excerpt 19:

*Interpreters should cooperate and be capable of translating the speech of practitioners and patients accurately and completely, without selectively interpreting or freely altering what is being said. (Practitioner J)*

Second, they believed an ideal interpreter should have sufficient health literacy, be aware of the systematic and conceptual differences between TCM and Western medicine, and be able to help patients appreciate these differences during interpreting. Excerpt 20 reflects this phenomenon:

*TCM has its own set of discursive system, which differs significantly from ordinary everyday language or the language of Western medicine. Ideal Interpreters should be aware of these differences, because if interpreters don't understand the clinical logic underlying TCM discourse, it is difficult to truly convey the intended meaning of practitioners. They should also have the ability and strategies to make patients aware of these differences and help patients truly understand our perspective. (Practitioner P)*

Third, practitioners maintained a cautious stance toward AI. Some believed that although AI could facilitate basic communication, it should be limited primarily to a supportive role rather than serving as the primary means in clinical communication. This is expressed in Excerpt 21:

*A real human interpreter who understands TCM is better than any overhyped AI tools. AI can be used, but only as an auxiliary support; it should not take over the interaction or dictate the communication process. (Practitioner N)*

Fourth, practitioners also suggested that specialized AI tools for TCM healthcare interpreting should be developed. This is presented in Excerpt 22:

*Relevant parties should cooperate to develop an app or AI tool for TCM healthcare interpreting. Ideally, it would also embed cross-references between TCM and Western medicine and offer concise explanations, through this way, it can also supplement patients with basic TCM knowledge and facilitate their understanding during interpretation. (Practitioner T)*

Last, practitioners reported that the communication challenges in cross-linguistic TCM clinical practice couldn't be resolved solely through individuals, but required long-term support from hospitals, training systems, and policy support. This is demonstrated in Excerpt 23:

*This issue goes beyond any individuals' capability; actually, it requires a top-down approach: policymakers, trainers and medical institutions should work together. Policymakers should confront the problem and issue policies to support academic institutions train professional TCM healthcare interpreters and require medical facilities to hire them. (Practitioner K)*

## 4 Discussion

The study reinforces previous research showing that untrained *ad hoc* interpreters tend to omit, summarize, or replace expressions when conveying information, which may negatively affect clinical diagnosis and treatment (6), and this phenomenon becomes more pronounced in contexts involving TCM syndrome differentiation, symptom descriptions, or emotional expressions, reflecting ad hocs' lack of professional ethics and insufficient interpreting competence. The involvement of AI does not fully resolve this problem, AI remains limited in accurately conveying TCM concepts and context-dependent clinical information, suggesting that findings reported in Western medical settings (24, 25) also apply to TCM consultations. One possible explanation lies in the limited availability of high-quality multilingual TCM corpora for model training: high-quality multilingual parallel corpora containing authentic TCM consultations remain scarce, and existing datasets are dominated by certain high-resource language pairs (e.g., English–Spanish) or common medical specialties (26). It is worth emphasizing that verbal information is not the only source of information in TCM encounters, paralinguistic and non-verbal information also contribute to provider-patient communication (27); importantly, the medical history, symptom descriptions and treatment preferences are primarily conveyed through language. Therefore, omission and errors in information transmission may not only result in the loss of clinically relevant information, but may also directly affect the clinical decision-making. It does not necessarily mean that TCM care cannot be delivered due to language barriers, but in the absence of effective language support, the risk of misunderstandings and clinical uncertainty would rise. Accordingly, providing effective language support not only concerns communication quality, but also patients' understanding of medical information and access to safe healthcare.

Since TCM diagnosis and treatment rely heavily on syndrome differentiation and holistic narration, cross-linguistic TCM medical encounters not only involve language barriers but also cognitive conflicts between TCM and Western medicine systems stemming from fundamental differences in the epistemological foundations of TCM and Western medicine. Grounded in traditional Chinese philosophy, TCM relies on holistic thinking, syndrome differentiation, and experiential knowledge rather than standardized and quantitative biomedical measurements (15). Many TCM concepts therefore are abstract, metaphorical, and highly context-dependent (16). Moreover, because these concepts are embedded in a conceptual system that differs substantially from that of Western biomedicine, many core TCM terms lack direct English equivalents (28). To tackle this challenge, some organizations, including WHO (World Health Organization) and WFCMS (World Federation of Chinese Medicine Societies) have promoted the standardization of TCM terminology partly by drawing on biomedical terminology (29). Although these translations achieve lexical equivalence, they often fail to achieve communicative equivalence and are regarded as a necessary compromise (30). The rapid evolution of AI has enabled *ad hoc* interpreters to quickly access the apparent biomedical equivalents for TCM terms, which may also flatten TCM concepts into biomedical labels, shrouding their broader theoretical and cultural meanings (31), and induce international patients with limited TCM literacy read these translated terms through familiar biomedical frameworks. Thus, a misalignment emerges between practitioners and international patients where the latter may misread the former's words and fail to appreciate the syndrome differentiation and treatment underlying practitioners' rationales. The “*ad hoc*+AI” mode accordingly facilitates semantic transfer rather than communicative equivalence, leaving the cognitive gap between TCM and Western medicine mostly unabridged.

The “*ad hoc* + AI” mode not only introduces additional layers of mediation into clinical communication but also reshapes existing power relations and trust dynamics. In conventional TCM clinical encounters, communication occurs through direct practitioner–patient interaction. However, with the involvement of *ad hoc* interpreters and AI, practitioners and patients' statements cannot be conveyed directly to each other but are mediated through *ad hoc* interpreters and AI. Interpretive authority is therefore redistributed among practitioners, *ad hoc* interpreters, AI, and patients. Previous studies have shown that *ad hoc* interpreters may filter, reorganize, or substitute information during medical encounters, thereby undermining physicians' informational authority (22, 32). Our findings suggest that the incorporation of AI further amplifies this tendency. Given *ad hoc* interpreters' heavy reliance on AI and their limited ability to critically evaluate AI-generated outputs, they increasingly assume a gate-keeping role in mediating clinical information. As a result, practitioners reported a diminished sense of control over clinical communication, as they were unable to determine what information ultimately reached patients or how it was transformed during transmission. AI support further strengthens *ad hoc* interpreters' influence over information transmission, reinforcing practitioners' concerns about communication accuracy and trust, thereby corroborating previous studies. While healthcare providers expressed concerns of AI-generated interpreting, patients generally reported high satisfaction (11). Our study yields a similar pattern, practitioners in this study perceived that many international patients tended to place considerable trust in AI-generated translations, partly because they interpreted through familiar biomedical frameworks. Moreover, due to concerns that ad hoc interpreters and AI may leak sensitive information, patients were inclined to withhold relevant details and limit discussion (22, 33, 34). Our findings further suggest that privacy concerns influence not only patients' willingness to disclose sensitive information but also practitioners' communication strategies. Practitioners reported deliberately reducing discussion of sensitive issues, partly because of privacy concerns and partly because of a perceived loss of control over how such information would be conveyed.

Previous studies have largely treated language barriers as a key obstacle to effective communication and quality care (6, 35). Our findings further suggest that persistent communication difficulties may also reshape practitioners' clinical decision-making. Some practitioners reported that when treating international patients, they often reduced explanations of TCM theories and actively avoided concepts that were likely to cause misunderstanding. As a result, the educational function of TCM consultations—where diagnosis and communication help patients understand TCM views of the body and illness (36)—is also weakened. International patients are hence often limited to relatively shallow, symptom-driven, and outcome-focused information, hindering their ability to truly grasp the holistic logic embedded in TCM syndrome differentiation. Such obstacles compromise not only the communication efficiency, but also the conveyance of TCM knowledge and the cultural and theoretical underpinnings of TCM. Meanwhile, communication constraints in turn limit the depth of syndrome differentiation and treatment. Individualized diagnosis in TCM is based on the integration of the four diagnostic methods—inspection, listening and smelling, inquiry, and palpation—among which inquiry plays a central role in understanding patients' subjective experiences and lifestyle factors and effective inquiry relies on in-depth, continuous, and high-quality doctor–patient communication (37). However, under the “*ad hoc*+AI” mode, due to high communication costs, a high risk of information distortion, and patients' limited comprehension ability, practitioners find it difficult to conduct in-depth inquiry in the same way as when treating Chinese patients, and are therefore compelled to rely more heavily on inspection, listening and smelling, and palpation to maintain basic syndrome differentiation, thereby limiting the completeness and precision of syndrome differentiation. This also explains why some practitioners report finding it difficult to develop truly individualized treatment plans for international patients. At a deeper level, such long-term communicative compromises may further affect practitioners' professional experience and professional identity. Providers' professional identity is closely tied to the ability to apply their professional knowledge and have it understood and accepted by patients (38). However, under conditions of prolonged cross-linguistic communication barriers, practitioners not only find it difficult to fully implement the diagnostic and therapeutic logic of TCM, but must also continuously cope with the frustration associated with repeated misunderstandings, repeated explanations, and communication breakdowns. Over time, some practitioners exhibit clear feelings of exhaustion toward cross-linguistic clinical encounters and even report feelings of fatigue and emotional burden when interacting with international patients. If such communication challenges persist, they may gradually reduce practitioners' willingness to engage in cross-cultural clinical practice and pose challenges to the sustainable development of intercultural TCM services.

Research has identified the limited access to professional interpreting services as a persistent challenge in cross-linguistic and cross-cultural healthcare encounters (39, 40). The accessibility and affordability of *ad hoc* interpreters (41) and AI tools (11) contribute to their widespread use despite their recognized limitations. When a substitute solution persists over a long period, the perceived urgency for institutional professionalization is often weakened (42). This study corroborates these findings by revealing that although most practitioners clearly acknowledge the drawbacks of “*ad hoc*+AI” mode, it is still being widely employed in healthcare practice. The prevalence of this mode seems to stem from practical necessity more than trust in its effectiveness. This trade-off may weaken the impetus for healthcare institutions to fund in professional interpreting services, thereby reinforcing a cross-linguistic healthcare model that favors basic information exchange at the expense of deeper understanding. Overall, “*ad hoc* + AI” mode ought to be considered as a pragmatic short-term solution rather than an optimal interpreting arrangement.

Practitioners' vision of ideal interpreting services transcends “accurate interpreting”. They anticipate services not only to convey information precisely and thoroughly, but also to enable cross-cultural communication, enhance international patients' understanding of TCM, and help them understand the conceptual framework underlying TCM. This echoes previous findings that healthcare interpreters function not only as linguistic mediators but also as cultural intermediaries (43–45). Most practitioners do not entirely reject the practical value of AI in cross-linguistic TCM consultations. Instead, they tend to attribute its deficiencies in meeting the demands of TCM practice to limited understanding understanding of the TCM knowledge system and insufficient capacity to interpret culturally embedded TCM concepts. Practitioners' expectations of AI interpreting tools are not constrained to the mere production of target-language text, but rather extend to an intelligent support system capable of providing contextualized explanations of relevant concepts by helping patients navigate between TCM and Western medical paradigms. This suggests that the demand for language services in cross-cultural TCM healthcare has moved beyond mere “information transfer” toward “interpretive support”. Practitioners also noted that this problem cannot be resolved solely by individual practitioners, *ad hoc* interpreters, or general-purpose AI tools, and instead requires a more stable and professional interpreting services. This aligns with existing research emphasizing the need for institutionalized language support and professional training systems in cross-cultural healthcare (22, 32).

Our findings suggest a need for professional interpreting services rather than relying solely on *ad hoc* interpreters or AI translation tools during consultations with international patients. Relevant educational institutions should train qualified TCM medical interpreters. Medical facilities ought to give priority to professional interpreting services whenever conditions permit; where *ad hoc* interpreters have to be employed, they must complete essential prerequisite training in interpreting ethics and role boundaries and be oriented to recognize the supportive not primary role of AI tools in medical consultations. Healthcare institutions should also provide TCM health literacy education as an essential service for international patients, offering conceptual explanations and theoretical clarifications as a part of clinical to enhance patients' understanding of the basic concepts, treatment logic and goals of their treatment. Moreover, endeavors should be expanded to accelerate the evolution of AI translation tools tailored to TCM clinical settings, upgrading its performance to process TCM knowledge systems, syndrome differentiation, and cultural contexts. In the long run, China should train TCM practitioners with bilingual or multilingual competence who can communicate with international patients independently and promote international cooperation in TCM education to support the training of local TCM practitioners in other countries, thus achieve language concordant TCM care.

## 5 Conclusion

This research investigated TCM practitioners' perspectives on AI-assisted *ad hoc* interpreting in China. The findings indicate that while “*ad hoc*+AI” mode can partially alleviate cross-linguistic communication barriers and is frequently used in practice under current practical constraints, it still often falls short of achieving effective cross-linguistic clinical communication, thereby undermining information transfer, practitioner-patient trust, syndrome differentiation and treatment, while gradually eroding practitioners' professional identity over time. Ideal interpreting services should not only ensure accurate and complete transmission of information, but also facilitate international patients' genuine understanding of the basic concepts, treatment logic and goals of their treatment. Ensuring access to such interpreting services requires policy support and collaboration among relevant stakeholders.

Future investigations could: (1) expand to a broader range of cross-linguistic healthcare settings, incorporate the perspectives of multiple stakeholders such as patients, interpreters, and hospital administrators; (2) use quantitative research methods empirically examine the associations among information loss, practitioner-patient trust, and clinical decision-making; (3) compare different types of AI tools, especially general-purpose AI tools and TCM-specific AI tools, to assess their utility and limitations in TCM interpreting.

## Data Availability

The datasets are not publicly available due to ethical and confidentiality restrictions imposed by the Institutional Review Board (IRB). Anonymized data may be made available from the corresponding author on reasonable request and with the permission of the ethics committee. Requests to access the datasets should be directed to 20220005@bisu.edu.cn.
